# Trospectomycin in Acute Pelvic Inflammatory Disease: A Preliminary Report

**DOI:** 10.1155/S1064744997000355

**Published:** 1997

**Authors:** Ashwin Chatwani, Vani Dandalou, Ozgur Harmanli, Paul Nyirjesy

**Affiliations:** Department of Obstetrics, Gynecology, and Reproductive Sciences Temple University Hospital 3401 N. Broad Street Philadelphia PA 19140 USA

## Abstract

*Objective:* The purpose of this study was to compare the clinical efficacy and safety of intravenous trospectomycin to that of cefoxitin plus doxycycline in the treatment of women hospitalized with acute pelvic inflammatory disease (PID).

*Methods:* Thirty-nine patients admitted with a clinical diagnosis of an acute PID were enrolled in this prospective, single-blind study. Patients were treated with either intravenous trospectomycin, 500 mg every 8 h, or intravenous cefoxitin, 2 g every 6 h, plus oral or intravenous doxycycline, 100 mg every 12 h, in a 2:1 ratio. The patients were followed for clinical response and side effects. Both groups of patients were discharged on oral doxycycline for 10 days. Appropriate cultures were obtained before starting inpatient treatment, on completion of inpatient treatment, and at 2 follow-up visits.

*Results:* The overall success rate for trospectomycin was 95.6% and for cefoxitin/doxycycline was 91.6%. This difference was not statistically significant (*P* = 0.63). Trospectomycin was found to be effective against *Chlamydia trachomatis*.

*Conclusions:* Single-agent therapy with trospectomycin may be as effective as cefoxitin plus doxycycline in the treatment of women hospitalized with acute PID.


Infectious Diseases in Obstetrics and Gynecology 5:215–218 (1997)
© 1997 Wiley-Liss, Inc.

### Trospectomycin in Acute Pelvic Inflammatory Disease: A Preliminary Report

Ashwin Chatwani,* Vani Dandalou, Ozgur Harmanli, and Paul Nyirjesy

*Department of Obstetrics, Gynecology, and Reproductive Sciences, Temple University School of Medicine, Philadelphia, PA*

### Abstract

***Objective:*** **The purpose of this study was to compare the clinical efficacy and safety of intravenous trospectomycin to that of cefoxitin plus doxycycline in the treatment of women hospitalized with acute pelvic inflammatory disease (PID).**

***Methods:*** **Thirty-nine patients admitted with a clinical diagnosis of an acute PID were enrolled in this prospective, single-blind study. Patients were treated with either intravenous trospectomycin, 500 mg every 8 h, or intravenous cefoxitin, 2 g every 6 h, plus oral or intravenous doxycycline, 100 mg every 12 h, in a 2:1 ratio. The patients were followed for clinical response and side effects. Both groups of patients were discharged on oral doxycycline for 10 days. Appropriate cultures were obtained before starting inpatient treatment, on completion of inpatient treatment, and at 2 follow-up visits.**

***Results:*** **The overall success rate for trospectomycin was 95.6% and for cefoxitin/doxycycline was 91.6%. This difference was not statistically significant (*P* = 0.63). Trospectomycin was found to be effective against *Chlamydia trachomatis.***

***Conclusions:*** **Single-agent therapy with trospectomycin may be as effective as cefoxitin plus doxycycline in the treatment of women hospitalized with acute PID. Infect. Dis. Obstet. Gynecol. 5:215–218, 1997.** © 1997 Wiley-Liss, Inc.

Key words cefoxitin; doxycycline; *Chlamydia trachomatis*

Acute pelvic inflammatory disease (PID) is a polymicrobial infection.[1–4] The most frequent pathogens involved in this mixed infection include *Neisseria gonorrhoeae*, *Chlamydia trachomatis*, aerobic organisms such as streptococcal species, enterococci, *Escherichia coli*, and anaerobic bacteria such as *Bacteroides* spp. and *Peptostreptococcus* spp. Since therapy must be instituted before availability of microbiologic results, empiric treatment with broad-spectrum antibiotics is recommended as initial therapy. Cefoxitin is widely used for the treatment of acute PID because of its broad-spectrum activity. However, cefoxitin is ineffective against *C. trachomatis* and hence is often used with doxycycline. There are also recent reports of development of resistance to cefoxitin by some enterococcal and staphylococcal species.[5]

Trospectomycin sulfate is a novel broad-spectrum aminocyclitol antibiotic with activity against a majority of aerobes, anaerobes, *N. gonorrhoeae*, and *C. trachomatis*.[6–8] This study was undertaken to evaluate the safety and clinical efficacy of

Contract grant sponsor: Upjohn Co.

*Correspondence to: Dr. Ashwin Chatwani, Department of Obstetrics, Gynecology, and Reproductive Sciences, Temple University Hospital, 3401 N. Broad Street, Philadelphia, PA 19140.

Received 15 May 96
Accepted 17 March 97

trospectomycin as a single-agent therapy in the treatment of acute PID. Monotherapy has many advantages. First, and most important to the patient and physicians, is the fact that combination therapy involves the possible toxic effects of two or more drugs. Furthermore, there is an ease of administration with monotherapy. It can be less expensive because of the savings of not mixing antibiotics and using fewer intravenous tubings.

### MATERIALS AND METHODS

The clinical protocol of this study was approved by the Institutional Review Board of Temple University School of Medicine. Thirty-nine consecutive patients admitted to Temple University Hospital with a clinical diagnosis of acute PID were enrolled in this study. The diagnosis of acute PID was based on the presence of abdominal pain and tenderness, uterine tenderness, cervical motion tenderness, and adnexal tenderness. An additional requirement was an elevated temperature (>100.4°F) and/or an elevated white cell count (>10,000/mm$^3$) and/or an elevated erythrocyte sedimentation rate (>20 mm/h) and/or the presence of purulent vaginal discharge.

At the time of admission, the cervix was gently wiped with a sterile swab and then specimens were taken from the endocervix and placed on Thayer Martin medium for *N. gonorrhoeae* and Sheppard's 10B broth for *C. trachomatis*. Endometrial samples were taken using transcervical aspiration curette (Pipelle®, Unimar, Inc., Wilton, CT) under aseptic conditions and processed for *C. trachomatis* and *N. gonorrhoeae* in similar fashion. In addition, endometrial cultures were also obtained for *Mycoplasma hominis*, *Ureaplasma urealyticum*, facultative aerobes, and anaerobes. The organisms were isolated and their susceptibility testing for all 3 antibiotics was performed using National Committee for Clinical Laboratory Standards.[9,10] Minimum inhibitory concentration (MIC) of 64 g/ml or less was considered susceptible. The laboratory tests included complete blood count with differential count, urinalysis, studies for renal and hepatic functions, serologic test for syphilis, and urine pregnancy test.

Using a simple computer-generated randomization table, patients were allocated to trospectomycin vs. cefoxitin plus doxycycline in a 2:1 ratio. Following randomization, trospectomycin was administered intravenously, 500 mg every 8 h, or ce-

**TABLE 1. Patient characteristics**

| Variable | Trospectomycin (N = 26) | Cefoxitin/ doxycycline (N = 13) |
|---|---|---|
| Age (years) | 26.7 ± 7.5[a] | 26.1 ± 4.6 |
| Weight (lb) | 151 ± 42.4 | 141.1 ± 30.2 |
| Past history of PID | 3 | 2 |
| Past history of sexually transmitted disease | 16 | 6 |
| Admission white cell count | 16.3 ± 6.2 | 16.2 ± 6.5 |
| Admission temperature (°F) | 101.3 ± 1.2 | 101.2 ± 1.4 |
| Length of stay (days) | 5.2 ± 0.9 | 4.8 ± 0.7 |

[a]Mean ± SD.

foxitin was given at a dosage of 2 g every 6 h along with 100 mg of doxycycline, orally or intravenously. The patients were followed daily for temperature, abdominal tenderness, and pelvic tenderness. A clinical failure was defined as persistent elevation of temperature of greater than 100.4°F and/or worsening of signs and symptoms of acute PID in a patient who had received the study medications for a minimum of 48 h.

The hematologic studies were repeated every 48 h while the patients were hospitalized. The patients were discharged after a minimum of 4 days of inpatient treatment and had achieved complete resolution of signs and symptoms of acute PID. All the cultures were repeated at the time of discharge. The patients in both groups were given 10 days of oral doxycycline. Patients received follow-up visits 4–7 days and 21–28 days after the completion of the outpatient treatment. The laboratory tests were repeated at the first follow-up visit and cultures were repeated on both follow-up evaluations.

Statistical analysis was performed using the Mantel-Haenszel chi-square formula. When a cell value of less than 5 was encountered, a two-tailed $P$-value was obtained by means of the Fisher's exact test. For continuous variables, a $P$-value was calculated through a one-way analysis of variance of the means. $P < 0.05$ was considered significant.

### RESULTS

Of 39 patients enrolled in the study, 26 received trospectomycin and 13 received cefoxitin plus doxycycline. There was no statistical difference between the 2 groups with respect to age, weight, admission temperature, and admission white cell count (Table 1). The length of hospital stay for the

TABLE 2. Isolates at admission[a]

| Organisms | Trospectomycin | | Cefoxitin/doxycycline | |
|---|---|---|---|---|
| | Endomet | Endocx | Endomet | Endocx |
| *N. gonorrhoeae* | 12 | 17 | 6 | 3 |
| *C. trachomatis* | 3 | 3 | 2 | 1 |
| *M. hominis* | 13 | 18 | 8 | 10 |
| *U. urealyticum* | 14 | 20 | 8 | 10 |
| Gram-positive aerobes | | | | |
| *Streptococcus* spp. | 13 | | 4 | |
| *Enterococcus* spp. | 3 | | 2 | |
| *Staph. epidermidis* | 4 | | 1 | |
| Gram-negative aerobes | | | | |
| *E. coli* | 3 | | 3 | |
| *Enterobacter* sp. | 0 | | 1 | |
| Anaerobes | | | | |
| *Bacteroides* spp. | 10 | | 6 | |
| *Peptostreptococcus* spp. | 3 | | 1 | |
| *Gardnerella vaginalis* | 3 | | 2 | |

[a]Endomet, ; Endocx, .

trospectomycin group was 5.2 ± 0.9 days, while that for the cefoxitin/doxycycline group was 4.8 ± 0.7 days. This was not statistically different. Three patients in the trospectomycin group were omitted from the study; 2 patients received a non-protocol antibiotic for a positive syphilis test and 1 patient had recurrent gonococcal infection. One patient in the cefoxitin/doxycycline group was omitted because of recurrent gonococcal infection.

The overall clinical success for trospectomycin was 95.6% (22 of 23) and for cefoxitin/doxycycline was 91.6% (11 of 12). This difference was not statistically significant. One failure in each group responded to alternative treatment with the combination of ampicillin, gentamicin, and clindamycin.

Isolates on admission are presented in Table 2. In general, MICs for trospectomycin were comparable to cefoxitin, except for those to *C. trachomatis*. As expected, the chlamydial isolates were sensitive to trospectomycin and doxycycline, but resistant to cefoxitin. Despite increasing resistance of organisms usually involved in acute PID to doxycycline, the pathogens isolated in our series were all susceptible to this antibiotic.

The overall frequency of adverse events was not significantly different between the 2 groups. There were no serious side effects in either group. Minor side effects included vomiting (1 in the cefoxitin/doxycycline group and 2 in the trospectomycin group); indigestion (1 in each group), and headache (1 in the cefoxitin/doxycycline group and 2 in the trospectomycin group). There were no clinically significant drug-related changes in blood urea nitrogen, creatinine, or bilirubin. One subject in the trospectomycin group showed elevation of liver enzymes on the day following completion of the inpatient treatment. These enzymes returned to normal at the second follow-up visit. There were no cases of antibiotic-associated colitis.

### DISCUSSION

This is the first study to examine the use of trospectomycin in the treatment of acute PID. This study demonstrates that trospectomycin is as effective as cefoxitin/doxycycline combination for inpatient treatment of upper genital tract infections.

Selection of appropriate therapy for the treatment of polymicrobial upper genital tract infections requires an understanding of the spectrum of activity of available antimicrobial agents. Trospectomycin is a 6′ propyl analog of spectinomycin with unique pharmacokinetics and broad spectrum of activity. It is highly effective against anaerobes, gram-positive and gram-negative aerobes,[6–8] penicillin-sensitive and -resistant *N. gonorrhoeae*,[11] and *C. trachomatis*.[6,12] A single 250 mg intramuscular injection of trospectomycin has been shown to be as effective as a single dose of ceftriaxone in the treatment of uncomplicated gonococcal infection.[11] In vitro studies have demonstrated significant activity against *C. trachomatis* (MIC 3.1–12.5 μg/ml), which constitutes an important extension over spectinomycin spectrum.[6,12] Trospectomycin is highly effective against *M. hominis* and at least

2-fold more active than macrolides and tetracyclines against *U. urealyticum*.[13] Trospectomycin has a prolonged post-antibacterial effect and a long serum half-life, 2.18 h compared to 0.5–1.0 h for cefoxitin.[7,14] Trospectomycin exhibits in vitro and in vivo antimicrobial activity for up to 9 h or longer after single intravenous or intramuscular injection.[7,14] Therefore, this antibiotic can be administered 2 or 3 times daily. The replacement of an antibiotic with less frequent dosing can result in a 35% savings in the annual cost of administration of antibiotics.[15,16] All of the above characteristics make trospectomycin an ideal initial antibiotic for the treatment of acute PID.

Since trospectomycin is effective against *C. trachomatis*, it can be used as a single-agent therapy for acute PID. The advantages of single-agent therapy are avoidance of toxic effects of multiple drugs, fewer allergic reactions, and less drug interactions. Furthermore, monotherapy is easy to administer and less expensive, because of savings in terms of nursing time and pharmacy charges. The administration of trospectomycin 3 times a day makes it even more attractive than cefoxitin.

Even though we discharged the patients on oral doxycycline after receiving inpatient trospectomycin, results of in vitro microbial susceptibility testing suggest that this may not be necessary. All 3 patients with chlamydial infection had negative cultures at the time of their discharge. Trospectomycin was well tolerated with few drug-related side effects.

The results of this preliminary study suggest that trospectomycin may represent a potential single-agent therapy for acute PID. A much larger study is needed to establish the role of trospectomycin as monotherapy for upper genital tract infections, especially with *C. trachomatis*.

### REFERENCES

1. Paavonen J, Teisala K, Heinonen PK, Aine R, Laine S, Lehtinen M, et al.: Microbiological and histopathological findings in acute pelvic inflammatory disease. Br J Obstet Gynaecol 94:454–460, 1987.
2. Brunham RC, Binns B, Guijon F, Danforth D, Kossrim ML, Rand F, et al.: Etiology and outcome of acute PID. J Infect Dis 158:510–517, 1988.
3. Soper DE, Brockwill NJ, Dalton HP, Johnson D: Observations concerning the microbial etiology of acute salpingitis. Am J Obstet Gynecol 170:1008–1017, 1994.
4. Eschenbach DA, Buchanan TM, Pollock HM, Forsyth PS, Alexander ER, Lin J, et al.: Polymicrobial etiology of acute PID. N Engl J Med 293:166–171, 1975.
5. Hager WD, McDaniel PS: Treatment of serious obstetric and gynecologic infections with cefoxitin. J Reprod Med 28:337–340, 1983.
6. Zurenko GE, Yagi BH, Vavra JJ, Wentworth BB: In vitro antibacterial activity of trospectomycin (U-63366F), a novel spectinomycin analog. Antimicrob Agents Chemother 32:216–223, 1988.
7. Betty H, Yagi BH, Schaadt RD, Zurenko GE: The bacterial activity and post-antibiotic effect of trospectomycin. Diagn Microb Infect Dis 15:417–423, 1992.
8. Applebaum PC, Spangler SK, Jacobs MR: Susceptibility of 539 gram positive and gram negative anaerobes to new agents, including RP 59500, biapenem, trospectomycin and piperacillin/tazobactam. J Antimicrob Chemother 32:223–231, 1993.
9. National Committee for Clinical Laboratory Standards: Methods for Dilution Antimicrobial Susceptibility Tests for Bacteria That Grow Aerobically. 2nd edition. NCCLS Publication M7-T2. Villanova, PA: NCCLS, 1985.
10. National Committee for Clinical Laboratory Standards: Alternative Methods for Antimicrobial Susceptibility Testing of Anaerobic Bacteria. NCCLS Publication M17-P. Villanova, PA: NCCLS, 1988.
11. Mroczkowski TF, Millikan LE, Martin DH, Leonik KJ: Treatment of gonococcal infections with a single 250 mg intramuscular injection of trospectomycin sulfate vs. ceftriaxone sodium. Drugs Exp Clin Res 19:41–46, 1993.
12. Kuo CC, Grayston JT: In vitro drug susceptibility of chlamydial species strain TWAR. Antimicrob Agents Chemother 32:257–258, 1988.
13. Echainz AG, Conde GC, Juarez FL, Carnalla BN, Tamayo LE, Calderon JE: In vitro activity of several antimicrobial agent against genital mycoplasmas. Clin Ther 14:688–695, 1992.
14. Nichols DJ, Bye A, Novak E: Pharmacokinetics of trospectomycin sulfate in healthy subjects after single intravenous and intramuscular doses. Br J Clin Pharmacol 32:255–257, 1991.
15. Martinusen S, Chew D, Frighetto L, Bunz D, Stiver H, Jewesson PJ: Comparison of cefoxitin and ceftizoxime in a hospital therapeutic interchange program. Can Med Assoc J 148:1161–1169, 1993.
16. Wright DB: Antimicrobial formulary management: A case study in a teaching hospital. Pharmacotherapy 11: 27s–31s, 1991.
